# Precision in Practice: The Critical Role of Mesh and Procedure Type Specification in Urogynecological Surgeries and Research

**DOI:** 10.1007/s00192-024-05820-5

**Published:** 2024-05-29

**Authors:** Reut Rotem, Daniel Galvin, Yair Daykan, Sumaiya Al-shukaili, Barry A. O’Reilly, Orfhlaith E. O’Sullivan

**Affiliations:** 1 Department of Urogynaecology, Cork University Maternity Hospital, Wilton Road, Wilton, Cork Ireland; 2 Department of Obstetrics and Gynecology, Shaare Zedek Medical Center, affiliated with the Hebrew University School of Medicine, Jerusalem, Israel; 3 Department of Obstetrics and Gynaecology, Meir Medical Center, Kfar Saba, Israel; 4 School of Medicine, Tel Aviv University, Tel Aviv, Israel; 5 Obstetrics and Gynaecology Department, Urogynaecology, Royal Hospital, Muscat, Oman

**Keywords:** Urogynecology, Mesh, Mesh type specification, FDA warnings, Midurethral sling, Safety

## Abstract

Recently, the debate surrounding the use of mesh in urogynecological procedures has intensified, leading to FDA warnings and heightened safety concerns. This clinical opinion emphasizes the vital need to specify mesh types in these procedures, drawing attention to the risk profiles and clinical outcomes associated with various meshes and the procedures that utilize them. A significant issue identified in contemporary literature is the tendency to group diverse mesh types under the same umbrella, disregarding their unique characteristics and applications. We describe the range of mesh types, their application routes, and associated complications, highlighting the risks of this nonspecific approach to patient safety and informed decision making. We critically examine the generalization of mesh terminology in clinical and research dialogues. Concluding with specific recommendations for health care providers and researchers, the paper advocates for a more nuanced understanding and communication in the field, ultimately aiming to improve patient care and safety in urogynecological practice.

## Introduction

The utilization of surgical mesh in urogynecological surgeries, particularly for the treatment of stress urinary incontinence (SUI) and pelvic organ prolapse (POP), marks a significant advancement in modern gynecological practice. Introduced in early 1990s [1], the application of mesh, especially in cases of recurrent POP, has proven to be a cornerstone in surgical intervention, offering a durable and effective solution where traditional methods often fall short [2, 3]. For SUI, surgical mesh, commonly in the form of midurethral slings, has become the standard of care owing to its efficacy and the minimal invasiveness compared with previous surgical alternatives [3].

However, the journey of surgical mesh in medical practice has been fraught with complexities and evolving perspectives, especially regarding safety and efficacy. The US Food and Drug Administration (FDA) has played a crucial role in this evolution, particularly concerning transvaginal mesh used for POP repairs. Following reports of complications such as mesh erosion, pain, and infection, the FDA heightened its scrutiny and regulatory measures [4]. This culminated in the reclassification of transvaginal mesh for POP from a moderate-risk device (class II) to a high-risk device (class III), reflecting growing concerns about its safety profile. In contrast, the regulatory stance on mesh for SUI, particularly midurethral slings, has remained more favorable, given their established safety and efficacy profile [4].

In contrast to the FDA's measured approach, some countries have adopted more stringent policies regarding mesh use in urogynecological surgeries. To the best of our knowledge, only two countries—the UK and Ireland—have taken a decisive stance, implementing an ongoing “pause” in the use of all types of mesh for these surgeries [5]. In these nations, the use of surgical mesh, particularly for POP and SUI, has effectively been banned, highlighting a more conservative approach to its application in light of safety concerns.

This approach differs notably from the policies adopted in other countries, where the use of surgical mesh is still permitted, albeit under specific regulations and guidelines [6]. These regulations often emphasize stringent patient selection criteria, informed consent processes, and the necessity for surgeons to have specialized training in the use of these devices. This varied international landscape reflects a broader, ongoing debate within the medical community regarding the balance between the benefits and risks of the use of surgical mesh in urogynecological procedures. The reasons underlying the heterogeneity in approaches to mesh complications in different countries has not been explored in detail in the literature. We suggest that the medicolegal and regulatory environment in the UK and Ireland, where there is a state-funded national indemnity program and high litigation rates has resulted in a reluctance to lift the mesh “pause.”

## Importance of Mesh and Procedure Type Specification for Selection in Urogynecological Surgeries

### Mesh Types, Materials, and Design

The landscape of surgical meshes used in urogynecology is diverse, with variations in both materials and design significantly influencing their clinical application. The most commonly used mesh types are nonresorbable materials, such as polypropylene, chosen for their durability and strength. However, differences in biocompatibility among various materials can lead to varying patient outcomes [7]. This variance is crucial, as the interaction between the mesh material and human tissue can affect everything from the inflammatory response to the integration of the mesh into the surrounding tissue. Additionally, the design of the mesh—including aspects such as whether it is woven or nonwoven, its pore size, which can be classified using the Amid system [8], and its overall density (lightweight vs heavyweight)—plays a pivotal role in determining its suitability for specific clinical scenarios [7]. Crucially, the method of mesh application, whether transvaginal or transabdominal, has a profound impact on risk and efficacy profiles. Specifically, transvaginal meshes are associated with higher complication rates in POP surgeries, highlighting the importance of this factor in surgical decision making, compared with the lower risks of complications observed with transabdominal approaches and midurethral slings for SUI [9].

### Clinical Scenarios and Mesh Type Implications

#### Recurrent Pelvic Organ Prolapse

Rates of recurrence after a primary surgery vary, with reported rates reaching significant levels of up to 36% [10]. Addressing recurrent POP effectively demands a focus on preventing subsequent occurrences. In this context, surgical mesh, including mesh kits and inlay mesh, plays a pivotal role. Traditionally, mesh kits were positioned and anchored [11] subcutaneously, a method initially favored but later found to be more prone to infections. The evolution of these kits led to a shift toward transvaginal placement, significantly reducing risks of infection [11]. Modern mesh kits [12] tailored to specific POP types, include pre-cut meshes, along with necessary implantation tools. Inlay mesh [12], favored for its minimal invasiveness, is particularly suitable for less severe cases or when a conservative approach is warranted. These contemporary mesh systems are characterized by a reduced mesh quantity and less extensive fixation to native tissue, addressing past concerns about infection and integration. The incorporation of anchors in these systems is vital for optimal positioning and functionality, especially in cases of compromised tissue integrity [13].

Despite these advancements, the ongoing high rates of complications highlight the critical need for careful mesh type selection and patient-specific considerations [14]. Durable meshes with a lower risk of erosion are preferred, offering a robust long-term solution for those experiencing repeated prolapses [15].

#### Stress Urinary Incontinence

The introduction of midurethral slings, particularly those made from polypropylene mesh, has revolutionized the treatment of SUI. Offering a minimally invasive yet highly effective solution, these slings have become a preferred option for many clinicians owing to their durability and lower risk profile [16]. Prior to the development of midurethral slings, transabdominal surgeries such as Burch colposuspension and rectus pubovaginal slings were common, but these more extensive surgeries often resulted in unsatisfactory outcomes, largely because of the high complication rates, including voiding difficulties [17].

Mini slings, notable for their shorter length and single-incision requirement, are aimed at reducing complications associated with longer sling systems [18]. Although they are effective for many patients, their suitability depends on individual patient factors and the severity of SUI [19]. However, it is important to note that mini slings have not been universally endorsed and are not considered the mainstay of treatment worldwide.

The midurethral sling procedures, tension-free vaginal tape (TVT) and transobturator tape (TOT), referred to as retropubic and transobturator slings respectively, differ in anatomical positioning and risk profiles. The retropubic TVT places mesh tape under the urethra via small incisions and can be performed with either an upside-down or downside-up approach. The TOT routes tape through the obturator foramen and can be done in an outside-in or inside-out manner [20]. Each technique, and even the specific approach within these techniques, carries a distinct risk profile [21].

#### Sacrohysteropexy/Sacrocolpopexy

In recent years, sacrohysteropexy for uterine preservation has gained prominence, particularly among younger women who have yet to complete their family, or those choosing to retain their uterus for reasons of personal preference [22]. Sacrocolpopexy remains the gold standard procedure for vaginal vault prolapse and has the highest rates of patient satisfaction and lowest rate of recurrence compared with nonmesh and transvaginal means of vault support such as sacrospinous fixation [23]. A notable variability exists in sacrocolpopexy techniques, even among seasoned practitioners [24]. This variability spans different mesh types, the number and type of sutures used, suture placement, and dissection techniques. This diversity underscores the importance of a comprehensive evaluation to understand how these variations affect clinical outcomes. Accurate documentation and reporting of mesh types in these surgeries are necessary for evaluating risk profiles and complications effectively.

## Impact of Mesh and Procedure Type Heterogeneity and Ambiguity

Mesh type ambiguity in published research has a profound impact in both clinical practice and further research; a lack of specificity in identifying mesh types can lead to a range of consequences. In clinical practice, ambiguity can result in the selection of inappropriate mesh types, potentially leading to higher complication rates and adverse patient outcomes. It can also hinder effective patient counseling, joint decision making and informed consent, as patients may not be fully aware of the specific risks and benefits associated with the mesh used in their surgery. In research, the generalization of mesh types in studies can lead to misinterpretation of results, impacting joint decision making. Without clear documentation, it becomes challenging to accurately compare studies or aggregate data for meta-analyses, leading to potential biases in evidence-based practice. Recognizing these issues, our article delves into the necessity of adopting standardized, precise terminology and detailed reporting of mesh procedures to mitigate these challenges, thereby enhancing the accuracy of clinical decision making and the quality of urogynecological research.

## Recommendations for Practitioners and Researchers

### For Practitioners

#### Education and Training

Practitioners have an obligation to maintain a high level of education and training prior to performing or offering mesh procedures to their patients. Practitioners should be specifically trained in the comprehensive assessment and management of urogynecology patients, including history, physical examination, and urodynamic assessment. They should also have completed a comprehensive surgical training program, be competent in performing mesh-related procedures, and managing common complications. Ideally, this education should be achieved through formal subspeciality training in an accredited fellowship program.

#### Informed Patient Discussions

Practitioners have a crucial role in ensuring patient safety and satisfaction in urogynecological surgeries that use surgical mesh. It is vital to conduct informed discussions with patients, detailing the various mesh types (native, biological graft, synthetic) and clearly outlining the risks and benefits of each type. It is important to tailor the explanation of risks to the individual patient's circumstances, health literacy level, and personal concerns. Incorporating decision aids, such as brochures, videos, or digital applications, that provide visual and easily digestible information about the procedures, potential risks, and outcomes, may enhance patient comprehension and engagement in decision making. All the above-mentioned should facilitate joint decision making. Emphasizing precision and a patient-centered approach in these discussions is key to optimizing patient outcomes and satisfaction.

#### Risk Communication Per Route

It is important to clearly communicate the risks associated with different surgical routes: vaginal, midurethral sling (MUS), and abdominal. It is also important to explain how these routes can influence the outcome and the likelihood of complications. A transparent discussion about the specific risks of each surgical route helps patients to make informed decisions and sets realistic expectations about the outcomes of surgery.

#### Mesh Type and Alternatives

It is important to discuss in detail the specific types of mesh used, especially focusing on the material and design, and how these factors may affect the success of the surgery and the patient's recovery process. Additionally, it is crucial to present alternatives to mesh, enabling patients to make an informed choice. Considering the past controversies surrounding mesh, it may be beneficial to refer to it using different terminology, such as “synthetic graft,” when communicating with patients. This approach acknowledges the benefits of the material while addressing the potential concerns associated with its reputation.

#### Continual Postoperative Support and Surveillance

Providing thorough postoperative care and implementing long-term surveillance protocols are critical to the management of patients who have undergone mesh procedures. Health care facilities should maintain a database of patients who received implantable health care devices in accordance with international standards. Patients should be advised on how to access care in the event of complications or concerns and should receive comprehensive written information in this regard.

### For Researchers

Researchers have the responsibility to present clear and detailed information in their studies.

#### Clarity in Reporting

It is recommended to stress the importance of declaring the mesh type and route used in the study. If possible, this information should be highlighted in the title or at least in the abstract, as many clinicians rely on these sections alone for quick reference.

#### Detailed Methodology

Researchers should ensure that the methodology section of the research comprehensively details the type of mesh used, its application method, and the rationale behind these choices.

#### Policy Proposals for Publications

It is recommended to advocate for policy changes in medical journals and governing bodies, mandating the explicit mention of mesh types and application routes in publications. This will aid a better understanding and applicability of research findings.

## Conclusion

The specificity in identifying and reporting mesh types and procedures is a pivotal aspect of both clinical practice and research in urogynecology. Addressing the current ambiguity in mesh type reporting is crucial for enhancing patient outcomes and bolstering the reliability of research data. There is a clear call to action: practitioners must commit to comprehensive patient education about the risks and benefits of various mesh types and surgical approaches. Similarly, researchers are encouraged to ensure precise, clear reporting in their studies, striving for precision and homogeneity in their data.

As the field of urogynecology evolves, the adherence to these standards of clarity and precision by clinicians and researchers becomes increasingly important. This commitment is essential not only for improving patient care but also for driving progress in the field of urogynecological surgery. The medical community should prioritize specificity and transparency in using surgical meshes to uphold the highest standards of patient care and contribute to the development of best practices in this specialty.

The controversy surrounding surgical meshes has led to global skepticism and a rift between the industrial and clinical communities. However, we believe it is incumbent upon practitioners to lead the way. By critically examining the facts and focusing on evidence-based medicine, we can overcome these challenges, promote medical advancement in a thoroughly transparent manner and continue to provide the best possible care for our patients.
